# Profile of clinical characteristics and serologic markers of sporadic hepatitis E in a community cohort study

**DOI:** 10.1080/22221751.2022.2140613

**Published:** 2022-12-18

**Authors:** Zi-Min Tang, Gui-Ping Wen, Dong Ying, Si-Ling Wang, Chang Liu, Wei-Kun Tian, Ying-Bin Wang, Mu-Jin Fang, Yu-Lin Zhou, Yun-Sheng Ge, Ting Wu, Jun Zhang, Shou-Jie Huang, Zi-Zheng Zheng, Ning-Shao Xia

**Affiliations:** a State Key Laboratory of Molecular Vaccinology and Molecular Diagnostics, National Institute of Diagnostics and Vaccine Development in Infectious Diseases, School of Public Health, Xiamen University, Xiamen, PR People’s Republic of China; b NMPA Key Laboratory for Research and Evaluation of Infectious Disease Diagnostic Technology, School of Public Health, Xiamen University, Xiamen, PR People’s Republic of China; c United Diagnostic and Research Center for Clinical Genetics, Women and Children's Hospital, School of Medicine and School of Public Health, Xiamen University, Xiamen, PR People’s Republic of China; d School of Life Sciences, Xiamen University, Xiamen, PR People’s Republic of China

**Keywords:** Hepatitis E virus, active hepatitis, antigen, serial serum samples, dynamic

## Abstract

Hepatitis E virus (HEV) is a pathogen of global significance, but the value of HEV-related markers in the diagnosis of hepatitis E remains controversial. Previous studies on hepatitis E profiles have been mainly cross-sectional and conducted among inpatients in large hospitals, and hepatitis E cases have been primarily defined by limited partial markers. In this community-based study, 4,110 active hepatitis cases from a population of nearly 600,000 were followed over 48 months and serial serum samples were collected. Both HEV pathogen (HEV RNA and antigen) and anti-HEV antibody markers were used to determine HEV infection status and the relationship between hepatitis and HEV infection. In total, 98 hepatitis E patients were identified and all available isolates from 58 patients belonged to HEV genotype 4. The mean age of the patients was 58.14 years, with an overwhelming proportion of males (70.4%). Hepatitis E accounted for 22.86% of active hepatitis cases with alanine aminotransferase levels ≥15.0-fold the upper limit of normal, suggesting the need to include HEV in routine testing for these patients. Ninety-two hepatitis E patients were positive for at least 2 of HEV antigen, anti-HEV IgM, and HEV RNA markers at presentation, and 90.22% of them were positive for HEV antigen and anti-HEV IgM. HEV antigen, HEV RNA, and anti-HEV IgM positivity were observed in 89.80%, 82.65%, and 93.88% of hepatitis E patients at presentation, respectively. However, only 57.14% of anti-HEV IgM positivity occurred in hepatitis E patients. These findings will advance our understanding of hepatitis E and improve diagnosis.

## Introduction

Hepatitis E virus (HEV) represents the most common cause of acute viral hepatitis worldwide [1]. The incidence of hepatitis E has surged considerably in recent years in many developed and developing countries. In Europe, the reported incidence has increased 10-fold, from 514 cases in 2005 to 5,617 cases in 2015 [2], and in China, the incidence of hepatitis E increased 8-fold between 1997 and 2014 [3]. Four major genotypes of HEV are pathogenic in humans and show notable differences in their epidemiological characteristics, clinical features, and hosts [1]. Genotypes 1 and 2 are associated with large-scale outbreaks in developing countries and circulate exclusively in humans. Genotypes 3 and 4 are zoonotic with animal reservoirs and are linked to sporadic cases worldwide. The presentation of HEV infection varies greatly [1], including asymptomatic and symptomatic infection. In symptomatic HEV infection, most individuals present with hepatitis.

Several laboratory blood tests have been developed to diagnose hepatitis E, including detection of HEV RNA, HEV antigen, anti-HEV IgM, anti-HEV IgG, and a ≥ 4-fold rise in anti-HEV IgG levels (abbreviated as anti-HEV IgG-R) [1,4,5]. However, the value of these HEV-related markers is debatable, as previous studies have provided conflicting conclusions regarding the clinical application of these markers [5–14]. Most of these studies have focused on either HEV pathogens (HEV RNA or HEV antigen) or anti-HEV antibodies (anti-HEV IgM, anti-HEV IgG, or anti-HEV IgG-R) [6–12]; additionally, some studies focused on HEV RNA and anti-HEV antibodies but not HEV antigen [5,13,14]. Recent studies have shown that individual HEV-related markers, especially HEV antigen, are valuable [6,15]. HEV antigen detection mainly involves recognition of the free secreted form of ORF2 protein, which is the major form of ORF2 protein in the serum and is not associated with the viral genome and virions [6,15]. Such nonvirion-associated viral antigens (e.g. hepatitis B surface antigen, HBsAg) in the serum have also been observed in hepatitis B virus (HBV) infection [16,17] and play an important role in the diagnosis of hepatitis B. Previous studies on HEV antigen have compared it with other HEV-related markers, which served as references [7–12] and most published data for HEV antigen in the diagnosis of hepatitis E have been obtained based on samples identified by HEV RNA [8,11,18]. To date, the value of HEV antigen in patients with hepatitis E has not been determined in a large-scale cohort study.

Many studies on the profile of sporadic hepatitis E have been conducted with inpatients from large hospitals [13,14,19–22], with some selection bias in the selection of study patients. Some studies conducted in Europe and the USA have been longitudinal [13,14], but most of the studies conducted in other countries have been cross-sectional and conducted in patients at presentation [19–22]. Additionally, most of these studies used either anti-HEV antibodies or HEV RNA as evidence for hepatitis E [13,14,19–22]. Overall, there is a paucity of data on the profile of hepatitis E defined using all HEV-related markers in blood tests. Thus, the disease burden of active hepatitis E needs further analysis, especially in community-based studies.

In two previous studies, we characterized the profiles of sporadic hepatitis E cases in a rural community from 2006 to 2007 [5,23]. However, these two studies had some limitations and did not provide sufficient evidence, as active hepatitis patients were screened only for anti-HEV antibodies and patients with anti-HEV antibody responses were then tested for HEV RNA [5,23]. Furthermore, rapid industrialization and socioeconomic development have occurred in China in recent years, and an HEV vaccine became commercially available in 2012. Under these circumstances, the prevalence of HEV is likely different from that before vaccination and is worth studying. In this study, we described the clinical and viral profiles of hepatitis E patients based on a community-based cohort and comprehensively analyzed the dynamics and value of anti-HEV antibodies and HEV pathogen markers during illness progression.

## Materials and methods

### Patients and samples

Between January 2013 and December 2016, patients with suspected hepatitis in Dongtai, Jiangsu Province, China, were identified and enrolled through a well-established active hepatitis surveillance system [23,24]. This hepatitis surveillance system encompassed all health care centers in the study area, including private and village clinics as well as hospitals in each township and in the city [23,24]. Serum samples were obtained from patients presenting at these centers with hepatitis-like symptoms of fatigue and/or anorexia lasting ≥3 days and alanine aminotransferase (ALT) levels were assessed. Patients with abnormal ALT levels (≥2.5 times the upper limit of normal (ULN)) were followed up, and follow-up serum samples were collected if possible. This study was approved by the Ethics Committee of the School of Public Health, Xiamen University. Written informed consent was obtained from all participants.

### Detection of HEV antigen, anti-HEV IgM and anti-HEV IgG

HEV antigen, anti-HEV IgM and anti-HEV IgG in serum samples were evaluated using commercial kits (Wantai, Beijing, China). The antigen detection kits were optimized as previously reported [10] and are used for research only. Anti-HEV IgG levels in each sample were measured using the World Health Organization (WHO) reference serum as previously described [25,26]. The detection limit of the anti-HEV IgG kit was 0.077 WHO units per milliliter (hereafter, WU/mL) [25,26]. If serum samples were negative for anti-HEV IgG, the anti-HEV IgG levels of these samples were artificially set to 0.0385 WU/mL. All serum samples were stored at −20 °C prior to detection.

### HEV RNA detection and genotype analysis

Serum samples from patients with detectable HEV antigen, detectable anti-HEV IgM or positive anti-HEV IgG-R were individually tested for HEV RNA using a previously reported real-time reverse transcriptase PCR (RT–PCR) method [10,27,28]. The RT–PCR cycle threshold (Ct) value of ≤38 was considered as positive. Serum samples testing positive for HEV RNA were further analyzed to identify the HEV genotype via nested RT–PCR, Sanger sequencing, and phylogenetic analysis as described previously [28]. DNA sequences were aligned with corresponding regions of sequences retrieved from the GenBank database and analyzed using Mega 7.0 with the neighbor-joining method.

### Detection of hepatitis A virus (HAV), HBV and hepatitis C virus (HCV)

Patients with active hepatitis were tested for anti-HAV IgM, HBsAg, anti- HBV core protein IgM (anti-HBc IgM), and anti-HCV antibodies at presentation using commercial kits (Wantai, Beijing, China). A diagnosis of hepatitis A, hepatitis B, or hepatitis C was indicated by a positive finding for anti-HAV IgM, anti-HBc IgM, or anti-HCV antibodies, respectively.

### Statistical analysis

Statistical analysis was performed using GraphPad Prism software and SPSS. The significance of differences was determined by the Mann–Whitney U test for continuous variables and Pearson’s chi-square test for categorical variables. All reported *p* values were two-sided, and significance was considered at a *p* value <0.05.

## Results

### Profile of active hepatitis patients during the observation period

**Figure 1.**
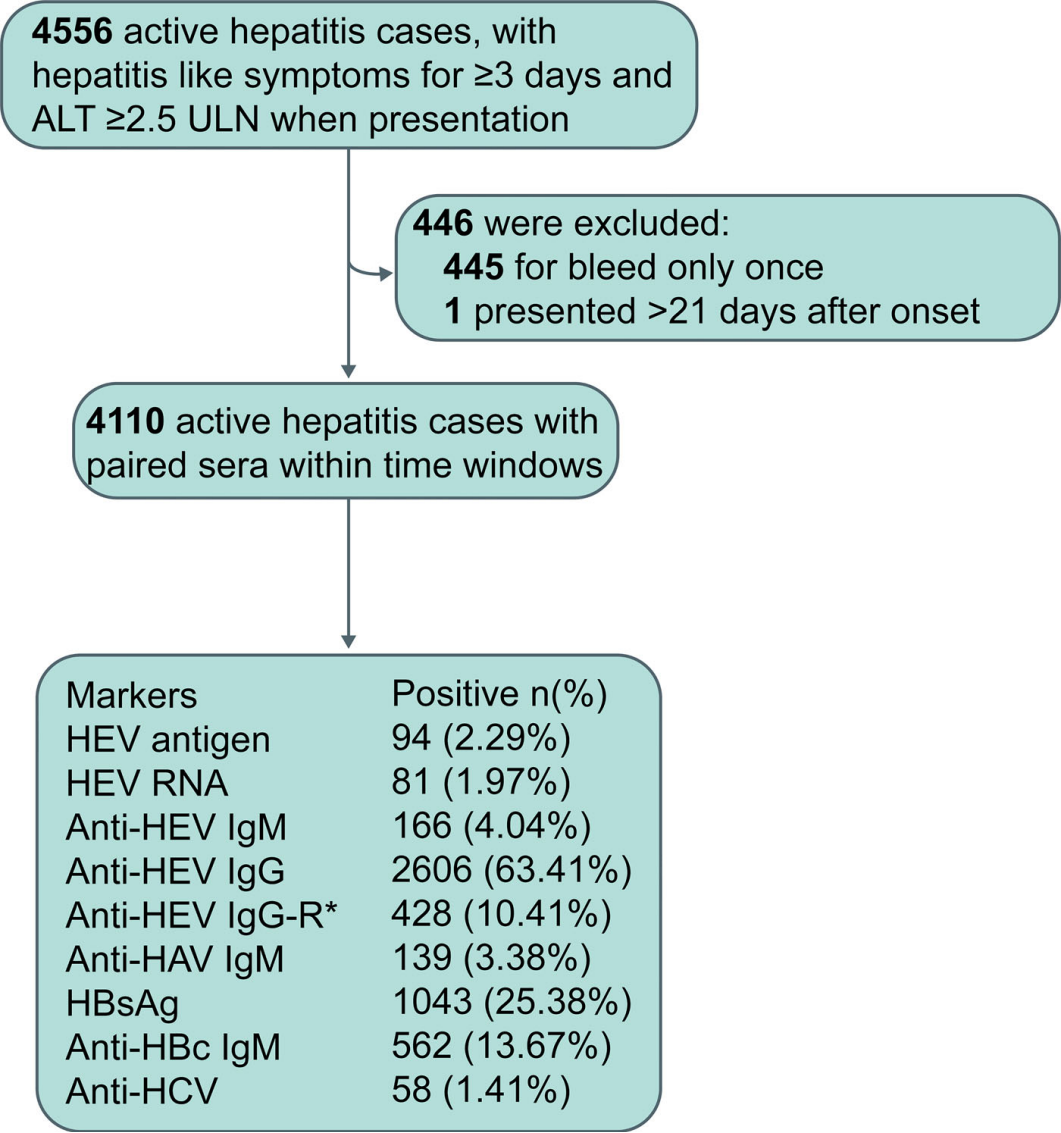
Profiles of active hepatitis patients during the period of observation. Hepatitis E virus (HEV)-related markers were measured in serial serum samples from active hepatitis patients; markers of hepatitis A virus (HAV), hepatitis B virus (HBV), and hepatitis C virus (HCV) were identified in the serum samples of patients at presentation. HBsAg: hepatitis B surface antigen. Anti-HBc IgM: anti-hepatitis B core protein IgM. Anti-HAV IgM: anti-hepatitis A virus IgM. Anti-HCV: anti-hepatitis C virus antibodies. Anti-HEV IgG-R: a ≥ 4-fold rise in anti-HEV IgG levels.

A total of 4,556 active hepatitis patients were observed over the 48-month study period; 4,110 presented within 21 days after onset and provided at least one another follow-up serum sample, these patients were included in the final analysis (Figure 1). The mean age of the 4,110 patients was 53.16 years and almost half (51.92%) of the patients were male. Among these 4,110 patients, the prevalence of HEV antigen, HEV RNA, and anti-HEV IgM was 2.29%, 1.97% and 4.04%, respectively. The prevalence of HBsAg and anti-HBc IgM was 25.38% and 13.67% at presentation, respectively. In total, 139 patients received a diagnosis of hepatitis A and 58 patients were considered to have hepatitis C.

### Diagnosis and exclusion of hepatitis E

**Figure 2.**
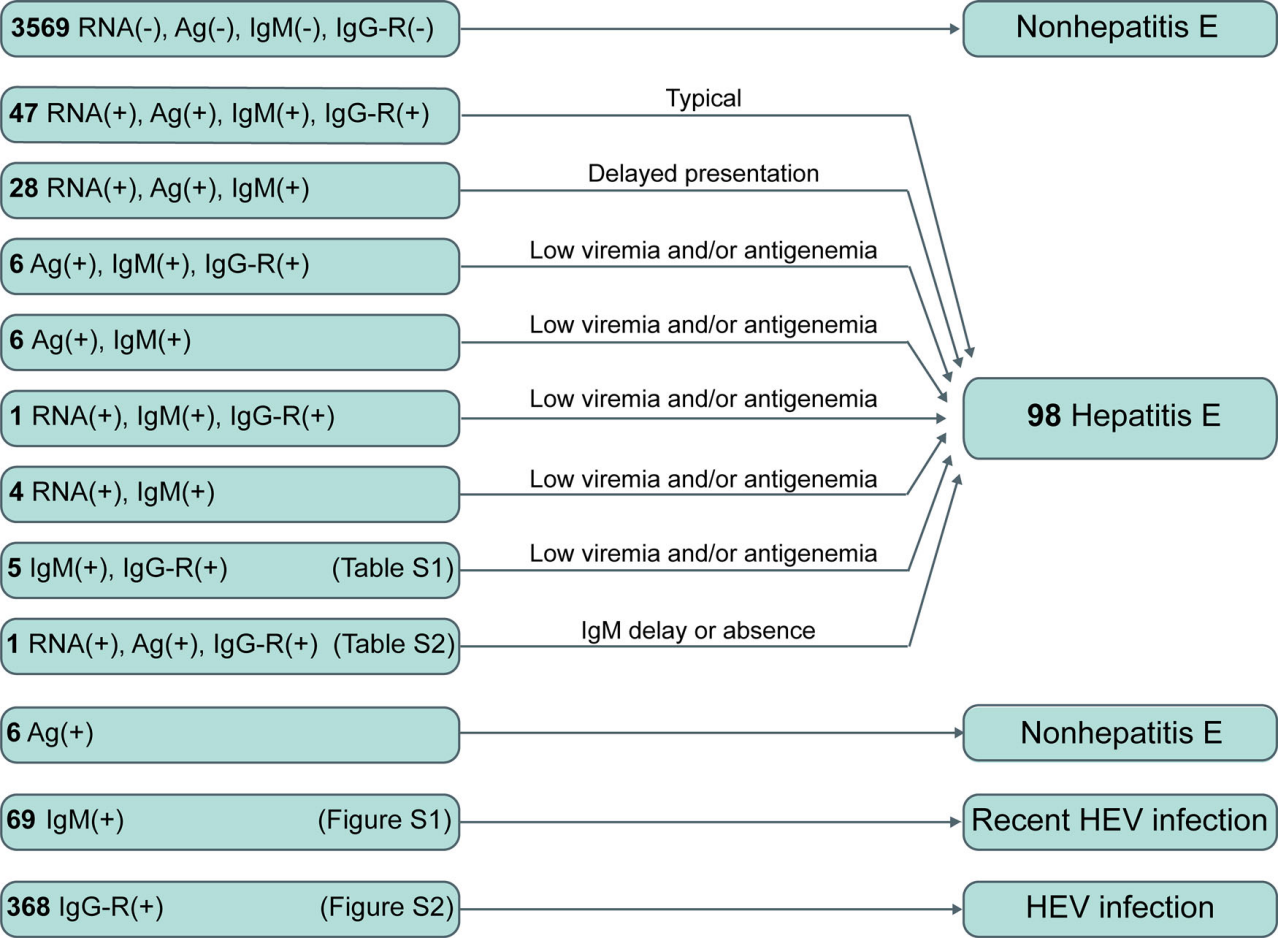
A schematic of how different groups of active hepatitis patients were defined. Among the 4,110 active hepatitis cases, 98 were identified as cases of hepatitis E. RNA: HEV RNA; Ag: HEV antigen; IgM: anti-HEV IgM; IgG-R: a ≥ 4-fold rise in anti-HEV IgG levels.

Among the 4,110 active hepatitis patients, 98 were identified as hepatitis E patients (Figure 2). Among 98 hepatitis E patients, 93 presented both the anti-HEV antibody response (anti-HEV IgM and/or anti-HEV IgG-R) and HEV pathogens (HEV RNA and/or HEV antigen). Seventy-five patients were positive for HEV RNA, HEV antigen, and anti-HEV IgM; of these, 47 displayed anti-HEV IgG-R. These 47 patients were considered typical cases of hepatitis E. Seventeen cases were positive for either HEV RNA or HEV antigen accompanied by anti-HEV IgM positivity. Five hepatitis E patients exhibited an obvious anti-HEV antibody response (positive for both anti-HEV IgM and anti-HEV IgG-R) without HEV pathogen markers in the serial serum samples (Table S1). Sixty-nine patients were positive for a single anti-HEV IgM. The dynamics of anti-HEV IgM and anti-HEV IgG in these 69 patients differed from those of the typical hepatitis E patients (Figure S1). This indicates that these patients may have recently been infected with HEV and that single anti-HEV IgM positivity may be due to the long-term persistence of anti-HEV IgM. Single-anti-HEV IgG-R positivity was observed in 368 patients, suggesting the presence of HEV infection in these patients. However, the levels of anti-HEV IgG in these patients were significantly lower than those in the typical hepatitis E patients (Figure S2). Thus, it was unclear whether the ALT abnormal of these patients were associated with hepatitis E. These 368 patients were considered to have an HEV infection.

### Characteristics of hepatitis E patients

**Figure 3.**
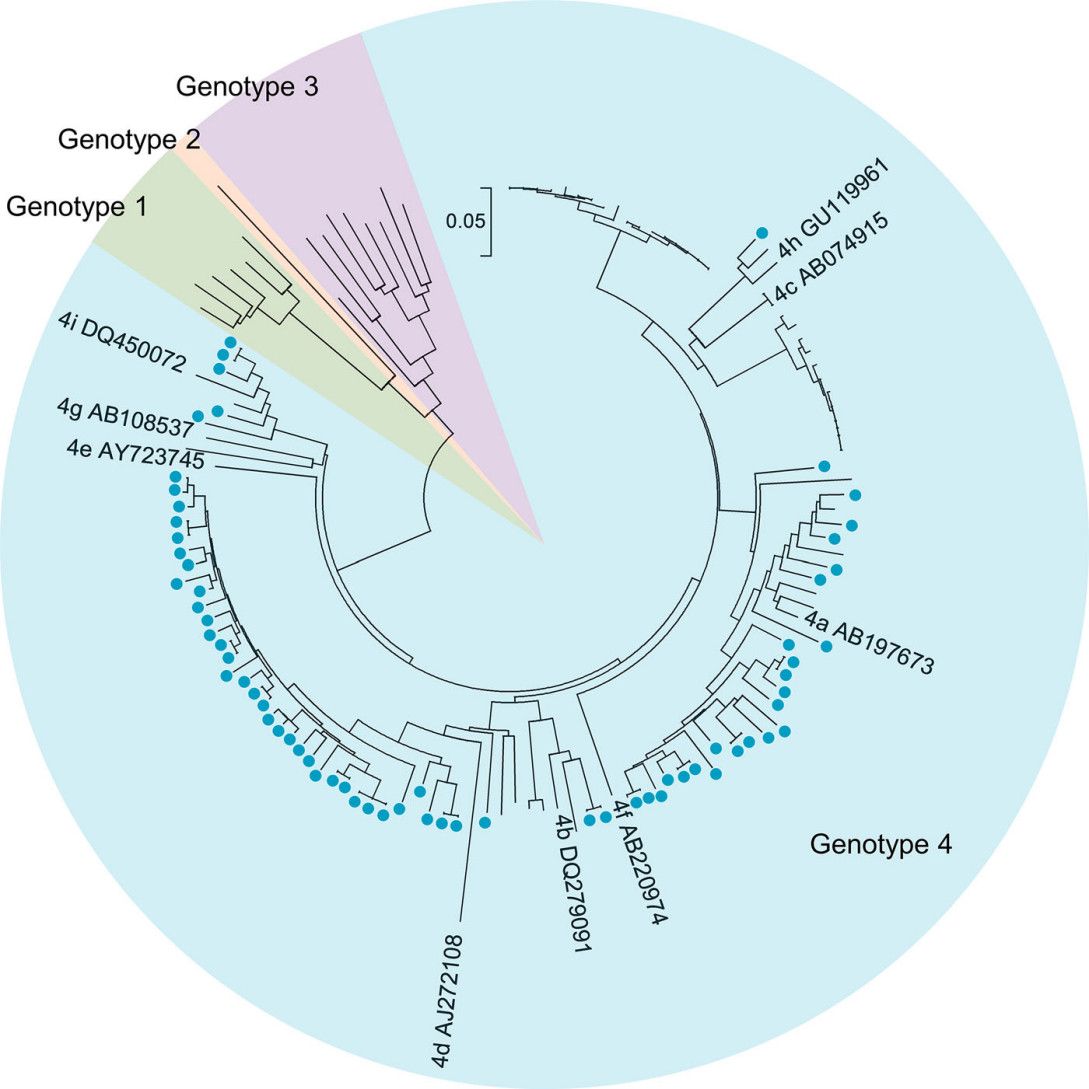
Phylogenetic tree based on partial open reading frame-1 nucleotide sequences from hepatitis E cases. Sixty-four sequences obtained from 58 hepatitis E cases are shown as blue dots. Viral sequences were obtained from serum samples collected from the 53 patients at presentation. Sequences were isolated from serum samples collected at presentation as well as from a second collection (in 4 patients) or from three serum samples (one patient). Accession numbers for the reference sequences are provided. The phylogenetic tree was constructed with the neighbor-joining method.

Among the 98 hepatitis E patients, 81 (82.65%) had detectable HEV RNA in one or more serum samples, indicating viremia among these patients. Hepatitis E patients with HEV viremia had significantly higher ALT levels than those without viremia (*p* = 0.035). HEV antigen was detectable in 88 (89.80%) patients, and anti-HEV IgM and anti-HEV IgG-R were found in 98.98% and 61.22% of hepatitis E patients, respectively. Sixty-four viral isolates from 58 patients were successfully sequenced. Phylogenetic analysis revealed that all viral isolates were HEV genotype 4 (Figure 3). A total of 53% of viral isolates were subtype 4d, and 34% were subtype 4a. Most (74.49%) hepatitis E cases were observed in winter and spring (from January to April) (Figure S3).

The clinical and laboratory profiles of patients with hepatitis E and other active hepatitis forms are summarized in Table 1. Hepatitis E patients had a mean age of 58.14 years and the majority (70.4%) of them were male. Hepatitis E patients were significantly older than patients with other viral hepatitis forms (*p* = 0.001) and hepatitis E-excluded patients (*p* = 0.002). Male hepatitis E patients showed significantly higher viral loads and longer symptomatic courses than female hepatitis E patients (Table S2). ALT levels in hepatitis E patients at presentation were significantly higher than those in hepatitis E-excluded patients and patients with other viral hepatitis forms (both were *p* < 0.001). The symptomatic course of hepatitis E cases lasted 57.66 ± 39.99 days, which was 3.10-fold and 2.87-fold longer than that observed in hepatitis E-excluded cases and cases of other viral hepatitis forms, respectively. Most illnesses in hepatitis E patients involved an uneventful recovery, but one death was recorded. Among the hepatitis E cases, 7 were comorbid with hepatitis B, and 2 were comorbid with hepatitis A.

**Table 1.** Characteristics of hepatitis E patients, other viral hepatitis patients, and active hepatitis patients.

Characteristic	All (*n* = 4110)	Hepatitis E (*n* = 98)	Hepatitis E-excluded (*n* = 4012)	Other viral hepatitis (*n* = 724)
Days after onset at presentation	4.65 ± 2.36	5.72 ± 3.23	4.62 ± 2.33	5.38 ± 3.09
Second serum collection (days after onset)	24.00 ± 10.43	24.31 ± 12.34	23.99 ± 10.38	25.16 ± 12.19
Sex (Male:Female)^#^	2134:1976	69:29	2065:1947*	428:296*
Age (years)^#^	53.16 ± 16.24	58.14 ± 13.67	53.04 ± 16.28*	53.35 ± 14.96*
Alanine aminotransferase level at presentation (ULN)^#^	6.65 ± 10.19	40.83 ± 33.52	5.82 ± 7.07*	8.46 ± 11.61*
Length of symptomatic course (days)^#^	19.72 ± 12.68	57.66 ± 39.99	18.78 ± 9.43*	20.06 ± 11.49*
Mean (95% CI) level of anti-HEV IgM at presentation (S/CO)	0.35(0.30–0.40)	8.61(7.71–9.50)	0.15(0.13–0.17)	0.11(0.08–0.14)
Mean (95% CI) peak level of anti-HEV IgG (WU/mL)	12.10(6.27–17.92)	386.24(199.18–573.30)	2.96(−0.52–6.43)	1.13(0.70–1.56)
Positive for HEV antigen, n (%)	94 (2.29)	88 (89.80)	6 (0.15)	1 (0.14)
Positive for HEV RNA, n (%)	81 (1.97)	81 (82.65)	–	–
Positive for anti-HEV IgM, n (%)	166 (4.04)	97 (98.98)	69 (1.72)	9 (1.24)
Positive for anti-HEV IgG, n (%)	2606 (63.41)	98 (100.00)	2508 (62.51)	432 (59.67)
Positive for anti-HEV IgG-R, n (%)	428 (10.41)	60 (61.22)	368 (9.17)	52 (7.18)
Positive for anti-HAV IgM, n (%)	139 (3.38)	2 (2.04)	137 (3.41)	137 (18.92)
Positive for HBsAg, n (%)^#^	1043 (25.38)	35 (35.71)	1008 (25.12)*	482 (66.57)
Positive for anti-HBc IgM, n (%)	562 (13.67)	7 (7.14)	555 (13.83)	555 (76.66)
Positive for anti-HCV, n (%)	58 (1.41)	0 (0.00)	58 (1.41)	58 (8.01)

#Differences between hepatitis E and hepatitis E-excluded/other viral hepatitis were calculated with the Mann–Whitney U test.

*Significantly different from hepatitis E patients (*P* < 0.05). Data are presented as the mean ± SD unless otherwise noted. CI denotes confidence interval. ULN: the upper limit of normal. WU/mL: WHO units per milliliter. S/CO: signal to cut off. A diagnosis of hepatitis A, hepatitis B, or hepatitis C was indicated by a positive finding for anti-HAV IgM, anti-HBc IgM, or anti-HCV, respectively. Active hepatitis patients who were positive for hepatitis A, hepatitis B, and/or hepatitis C and negative for hepatitis E were defined as having other viral hepatitis. Active hepatitis patients who were negative for hepatitis A, hepatitis B, hepatitis C, and hepatitis E were defined as indeterminate. HEV: hepatitis E virus. Anti-HEV IgG-R: a ≥ 4-fold rise in anti-HEV IgG level. HBsAg: the surface antigen of hepatitis B virus. Anti-HBc IgM: anti-core protein of hepatitis B virus IgM. Anti-HAV IgM: anti-hepatitis A virus IgM. Anti-HCV: anti-hepatitis C virus antibodies.

### Dynamics of HEV-related markers during disease progression

**Figure 4.**
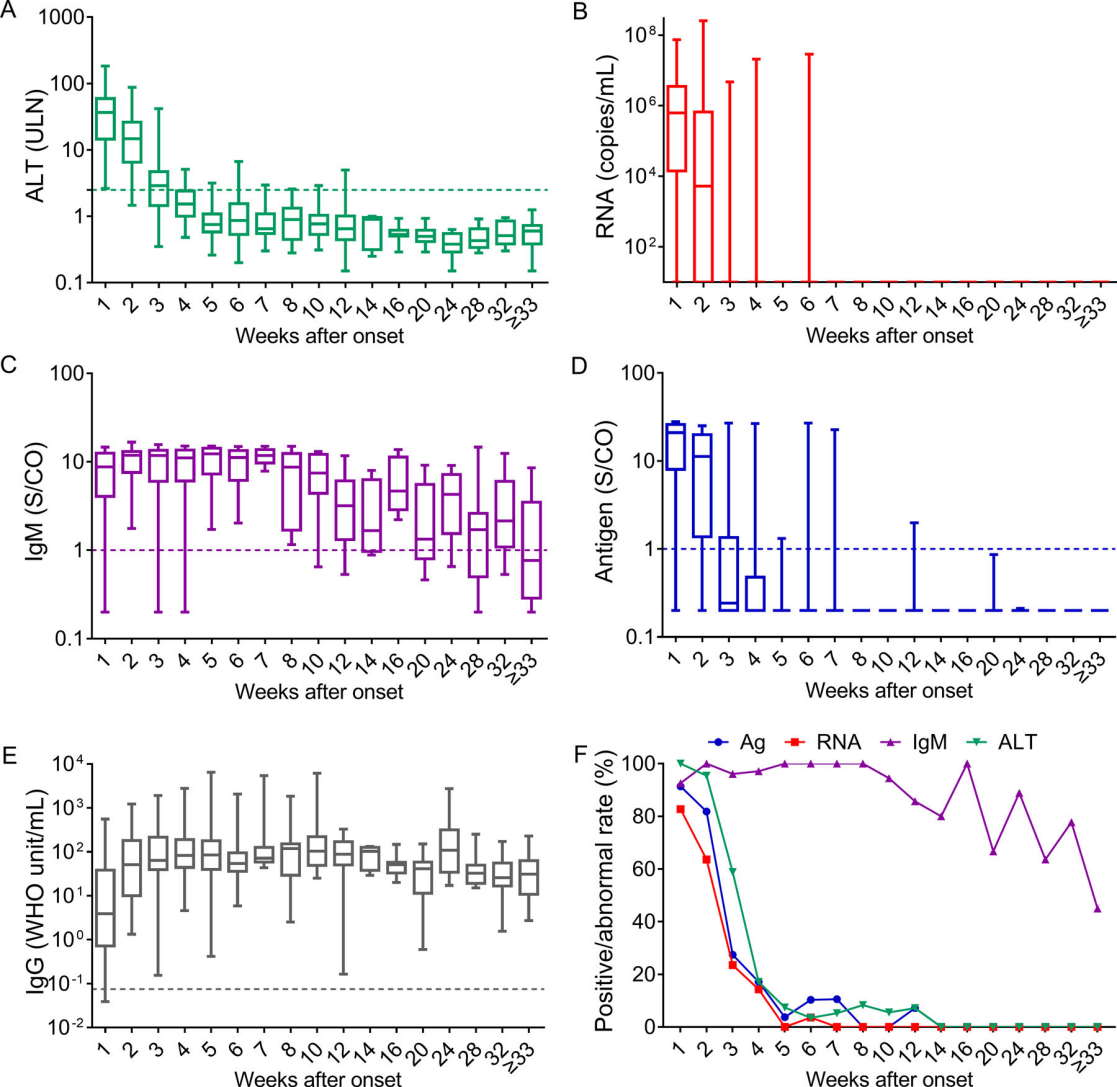
Dynamics of HEV-related markers in hepatitis E patients during disease progression. Serum samples were collected from 98 hepatitis E patients within the 1st week of symptom onset (*n* = 81) and in the following intervals after symptom onset, the 2nd week (*n* = 22), 3rd week (*n* = 51), 4th week (*n* = 35), 5th week (*n* = 27), 6th week (*n* = 29), 7th week (*n* = 19), 8th week (*n* = 12), 9–10 weeks (*n* = 18), 11–12 weeks (*n* = 14), 3–14 weeks (*n* = 5), 15–16 weeks (*n* = 8), 17–20 weeks (*n* = 9), 21–24 weeks (*n* = 9), 25–28 weeks (*n* = 11), 29–32 weeks (*n* = 9), and ≥33 weeks (*n* = 20). Levels of alanine aminotransferase (ALT) (A), HEV RNA (B), anti-HEV IgM (C), HEV antigen (D), and anti-HEV IgG (E) in the different serum sample intervals are shown in terms of the range (whiskers), interquartile range (boxes) and median (line within the boxes) values. The dotted lines represent the cutoff levels of ALT, anti-HEV IgM, HEV antigen, and anti-HEV IgG. (F) The positive rates of HEV antigen, HEV RNA and anti-HEV IgM and the rate of abnormal ALT levels in each group. ALT levels ≥2.5-fold the upper limit of normal (ULN) were considered abnormal in this study.

We further analyzed the dynamics of ALT, HEV antigen, HEV RNA, anti-HEV IgM, and anti-HEV IgG during disease progression (Figure 4). Overall, the levels of HEV RNA, HEV antigen, and ALT peaked within the 1st week after onset and declined thereafter. HEV RNA was detected in 82.72% (67/81), 63.64% (14/22), 23.53% (12/51), and 14.29% (5/35) of samples collected in the 1st, 2nd, 3rd, and 4th weeks after onset, respectively. HEV antigen was detected in 91.36%, 81.82%, 27.45%, and 17.14% of samples collected in the 1st, 2nd, 3rd, and 4th weeks after onset, respectively. The rates of abnormal ALT levels were 100.00%, 95.45%, 58.82%, and 17.14% in the 1st, 2nd, 3rd, and 4th weeks after onset, respectively. Anti-HEV IgM positivity was found in ≥92.59% of samples collected within four weeks after onset; HEV antigen and HEV RNA positivity were ≤10.53% and ≤3.57%, respectively, from 5 to 8 weeks after onset, and rates of abnormal ALT levels were ≤8.33% after 4 weeks after onset. Anti-HEV IgM was found in all samples collected from 5 to 8 weeks after onset and remained detectable in at least 63.64% of samples collected from 9 to 32 weeks after onset. Therefore, the duration of anti-HEV IgM appears to be much longer than that of HEV viremia, antigenemia and ALT abnormalities. Anti-HEV IgG levels presented an obvious increase within 4 weeks and were maintained at high levels even more than 33 weeks after onset.

### HEV-related markers in the first serum samples collected from hepatitis E patients and active hepatitis patients

**Figure 5.**
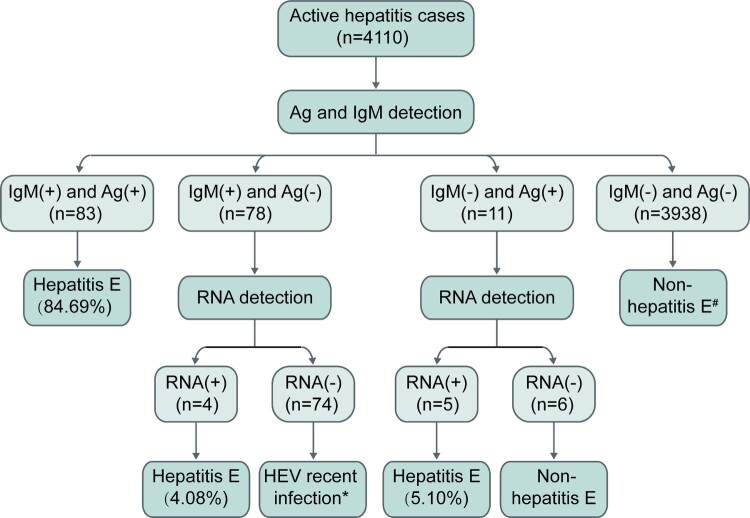
HEV RNA, HEV antigen, and anti-HEV IgM in the first serum samples collected from active hepatitis patients. ^#^ One hepatitis E patient showed HEV RNA positivity at presentation and anti-HEV IgM seroconversion and a ≥ 4-fold increase in anti-HEV IgG levels at follow-up. If HEV antigen and anti-HEV IgM were used as the first-line for the diagnosis of hepatitis E, this case would be considered non-hepatitis E at presentation. * Five hepatitis E cases presenting anti-HEV IgM and a ≥ 4-fold rise in anti-HEV IgG levels were identified to have a recent HEV infection at presentation and were confirmed as hepatitis E cases during follow-up. Ag: HEV antigen; IgM: anti-HEV IgM; RNA: HEV RNA.

In routine clinical practice, diagnosis based on a single sample is common. Therefore, we further analyzed HEV-related markers in patients at presentation. As shown in Figure S4, 92 (93.88%) hepatitis E patients had detectable anti-HEV IgM, and positivity for anti-HEV IgM was highest among HEV-related markers. A total of 161 active hepatitis cases were positive for anti-HEV IgM at presentation, but only 57.14% of these patients had hepatitis E (Figure 5), revealing the inaccuracy of using anti-HEV IgM alone for determining hepatitis E. HEV antigen positivity was observed in 89.80% of hepatitis E patients, and 93.62% (88/94) of patients positive for HEV antigen were hepatitis E patients. HEV RNA was detected in 82.65% of hepatitis E patients at presentation. These results suggest that if a single marker is used to determine hepatitis E, HEV antigen is the most appropriate.

From these results, it is obvious that using HEV RNA, HEV antigen or anti-HEV IgM alone to diagnose hepatitis E can lead to false negative and/or false positive results. However, combining multiple HEV-related markers might improve the accuracy of diagnosis. In this study, all hepatitis E patients were positive for anti-HEV IgM or HEV RNA and all but one hepatitis E patient were positive for anti-HEV IgM or HEV antigen. The first sample from 92 (93.88%) hepatitis E patients was positive for at least 2 of the HEV antigen, HEV RNA and anti-HEV IgM markers; 84.69% (83/98) of patients were positive for HEV antigen and anti-HEV IgM. Thus, given cost, expertise, and equipment requirements, we recommend that anti-HEV IgM and HEV antigen be used for first-line detection, with HEV RNA providing additional confirmation, for the clinical diagnosis of hepatitis E.

### Proportion of hepatitis E cases among active hepatitis cases

**Figure 6.**
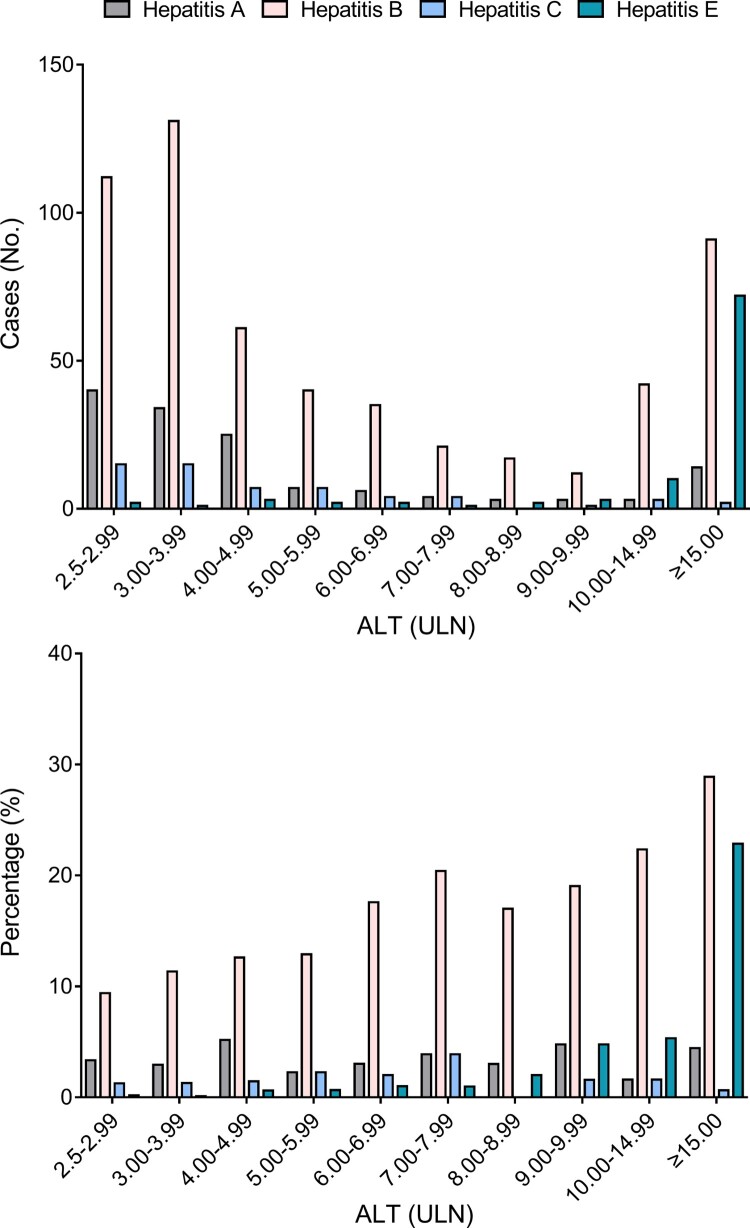
Rates of hepatitis E, hepatitis A, hepatitis B, and hepatitis C among active hepatitis patients with different alanine aminotransferase (ALT) levels. A diagnosis of hepatitis A was indicated by samples positive for anti-HAV IgM; a diagnosis of hepatitis B was indicated by samples positive anti-HBc IgM; and a diagnosis of hepatitis C was indicated by samples positive anti-HCV. ULN: the upper limit of normal.

This study adopted a low ALT limit for the definition of active hepatitis, facilitating detection of milder forms of hepatitis E. At presentation, 3.06% of hepatitis E patients had ALT levels <4×ULN, and 83.67% had ALT levels ≥10×ULN (Table S3). Nearly half of the hepatitis A and hepatitis B patients had ALT levels <4×ULN, and 12.23% of hepatitis A and 23.67% of hepatitis B patients had ALT levels ≥10×ULN. The proportion of hepatitis E cases increased drastically in active hepatitis cases with high ALT levels (Figure 6). Among active hepatitis cases with ALT levels <10×ULN at presentation, hepatitis E accounted for 0.09% to 4.76% and hepatitis B accounted for 9.39% to 20.39%. However, the proportion of hepatitis E cases (22.86%) among patients with ALT levels ≥15.0×ULN was 5.14-fold greater that of hepatitis A cases and comparable to that of hepatitis B cases (28.89%). These results suggest that hepatitis E is one of the main causes of active hepatitis in patients with ALT levels ≥15.0×ULN.

## Discussion

The dynamics of HEV-related markers during disease progression are vital for understanding active hepatitis cases and the selection of strategies to diagnose hepatitis E. However, these dynamics have not been fully explored, as previous studies have mainly focused on partial HEV-related markers or included only small numbers of cases (e.g. 16, cases) [5,10,18,29,30]. The dynamics of HEV-related markers during disease progression are described in detail in our study. Overall, abnormal ALT levels were associated with HEV antigen positivity and HEV RNA positivity. HEV antigen and HEV RNA were detectable in 81.82–91.36% and 63.64–82.72% of hepatitis E patients in the 1st and 2nd weeks after onset, respectively. While, anti-HEV IgM persisted for 8 months after ALT levels returned to normal, similar to observations in previous studies [5,10,29]. Additionally, we found that only 57.14% of anti-HEV IgM positivity was associated with hepatitis E patients. Therefore, we recommend that HEV RNA and HEV antigen, but not anti-HEV IgM, be used as markers for diagnosing hepatitis E within 2 weeks after symptom onset. We found that 58.82% of serum samples from hepatitis E patients displayed abnormal ALT levels at the 3rd week after onset. The positivity of HEV antigen, HEV RNA, and anti-HEV IgM in hepatitis E patients at the 3rd week after symptom onset were 27.45%, 23.53%, and 100.00%, respectively, suggesting that anti-HEV IgM is a better indicator of hepatitis E than HEV antigen and HEV RNA in this period. In the present study, a drastic decrease in positivity for HEV RNA with delayed collection of samples was observed, similar to previous studies [5,18,30,31]. In addition, the rate of HEV RNA positivity was lower than that of HEV antigen positivity throughout the progression of the disease. This finding may be due to the retrospective nature of this study, as all patients with active hepatitis were retrospectively tested for HEV-related markers. As HEV RNA may degrade during the process of transport and storage, HEV RNA positivity may have been underestimated. Currently, HEV RNA detection is not routinely performed in the clinic in China. Although some commercial HEV RNA detection kits are available in Europe [32], none of these kits are approved in China. Regardless, our results suggest that HEV RNA is a suboptimal marker for hepatitis E in retrospective studies and that HEV antigen testing is a useful diagnostic in resource-limited areas.

Many studies have explored the use of HEV antigen in the diagnosis of hepatitis E. The sensitivity of HEV antigen varies from 16 to 100% [6–12,21]. In this study, 89.80% of hepatitis E patients displayed HEV antigen positivity at presentation and 93.83% of HEV RNA-positive patients displayed detectable HEV antigen, suggesting that HEV antigen has high sensitivity for diagnosing hepatitis E, similar to previous studies [6,9,10]. We found that delays in specimen collection significantly decreased the rate of HEV antigen positivity although the rates of anti-HEV IgM and anti-HEV IgG positivity remained >95% from 2 to 8 weeks after onset. The low sensitivity of HEV antigen reported in previous studies [7,12] may be due to utilization of anti-HEV IgM or anti-HEV IgG positivity as the criterion for hepatitis E diagnosis and the timing of sample collection.

In this study, hepatitis E accounted for 2.38% of active hepatitis cases, a rate much lower than that observed in previous studies in the study area from 2006 to 2007 [5,23]. A phase III clinical trial of the hepatitis E vaccine was conducted in this area from 2007 to 2009, enrolling more than 100,000 participants among a half million registered individuals, with nearly half of participants receiving three doses of the hepatitis E vaccine [24,25]. This vaccine showed an efficacy of approximately 100% over a period of 19 months [25] and 93.3% over a period of 54 months [24]. Additionally, the local government launched a rural toilet improvement project in 2012 to improve the rural living environment [33]; hepatitis E prevalence might have gradually decreased with improvements in sanitary conditions. This finding is consistent with the fact that China has reduced HEV transmission by improving food and water hygiene as well as by administering HEV vaccinations [24,25,34].

Although a previous study reported no sex or age bias in HEV infection [26], we found significantly (*p* < 0.001) more hepatitis E patients were male (69/2134, 3.23%) rather than female (29/1976, 1.47%) in this study. A clinical feature of hepatitis E was an overwhelming preponderance of male patients in this study, consistent with previous studies [19,20,23]. Hepatitis E seems to be more severe in males than in females with a higher viral load and longer symptomatic course. These differences regarding sex may be associated with the lifestyle, environment or occupation [35,36]. Nearly half of the hepatitis E patients were ≥60 years old in this study, similar to previous studies in China and Egypt [20,21,23], and differing from findings in Italy [14] and the USA [13]. In this study, 22.86% of active hepatitis patients with ALT levels ≥15.0×ULN were hepatitis E cases. This emphasizes the need to include HEV screening in routine screens of active hepatitis patients with ALT levels ≥15.0×ULN. Additionally, HEV screening should receive more attention in winter and spring as this study and previous studies have found the most hepatitis E cases in these two seasons [19,23].

This study does have certain limitations. First, as all cases recruited were who attendees of community clinics, private clinics, or hospitals, this would result in the loss of cases that did not seek medical help. Second, HEV-related markers were detected in patients with active hepatitis at different time points with different times. In addition, HEV RNA testing was only performed on serum samples from patients who were positive for HEV antigen or anti-HEV IgM in any one serum sample, or positive for anti-HEV IgG-R in the serial serum samples, this may result in missing those case in which the early window may be positive for only the HEV RNA.

In summary, this study presented the demographic, clinical and molecular features of sporadic hepatitis E in a community cohort, providing a comprehensive profile of HEV-related markers during disease progression. Hepatitis E was found to be a major cause of active hepatitis in patients with severely elevated ALT levels. These findings advance our understanding of hepatitis E and may improve its diagnosis.

## Supplementary Material

Supplemental Material

## References

[1] Kamar N, Izopet J, Pavio N, et al. Hepatitis E virus infection. Nat Rev Dis Primers. 2017 Nov 16;3:17086. doi:10.1038/nrdp.2017.86 29154369

[2] The L. Growing concerns of hepatitis E in Europe. Lancet. 2017 Jul 22;390(10092):334. 10.1016/S0140-6736(17)31922-0 28745588

[3] Ren X, Wu P, Wang L, et al. Changing epidemiology of hepatitis A and hepatitis E viruses in China, 1990–2014. Emerg Infect Dis. 2017 Feb;23(2):276–279. doi:10.3201/eid2302.161095 28098527 PMC5324787

[4] Aggarwal R. Diagnosis of hepatitis E. Nat Rev Gastroenterol Hepatol. 2013 Jan;10(1):24–33. doi:10.1038/nrgastro.2012.187 23026902

[5] Huang S, Zhang X, Jiang H, et al. Profile of acute infectious markers in sporadic hepatitis E. PLoS One. 2010;5(10):e13560. doi:10.1371/journal.pone.0013560 21042408 PMC2958841

[6] Montpellier C, Wychowski C, Sayed IM, et al. Hepatitis E virus lifecycle and identification of 3 forms of the ORF2 capsid protein. Gastroenterology. 2018 Jan;154(1):211–223. doi:10.1053/j.gastro.2017.09.020 28958858

[7] Zhao C, Geng Y, Harrison TJ, et al. Evaluation of an antigen-capture EIA for the diagnosis of hepatitis E virus infection. J Viral Hepat. 2015 Nov;22(11):957–963. doi:10.1111/jvh.12397 25732029

[8] Behrendt P, Bremer B, Todt D, et al. Hepatitis E virus (HEV) ORF2 antigen levels differentiate between acute and chronic HEV infection. J Infect Dis. 2016;214(3):361–368. doi:10.1093/infdis/jiw161 27234418

[9] Geng Y, Zhao C, Huang W, et al. Detection and assessment of infectivity of hepatitis E virus in urine. J Hepatol. 2016 Jan;64(1):37–43. doi:10.1016/j.jhep.2015.08.034 26362822

[10] Wen GP, Tang ZM, Yang F, et al. A valuable antigen detection method for diagnosis of acute hepatitis E. J Clin Microbiol. 2015;53(3):782–788. doi:10.1128/JCM.01853-14 25540394 PMC4390668

[11] Tremeaux P, Lhomme S, Chapuy-Regaud S, et al. Performance of an antigen assay for diagnosing acute hepatitis E virus genotype 3 infection. J Clin Virol. 2016 Jun;79:1–5. doi:10.1016/j.jcv.2016.03.019 27038538

[12] Lapa D, Brega C, Mammone A, et al. Diagnostic performance of hepatitis E virus antigen assay in hepatitis E virus acute infection. New Microbiol. 2016 Oct;40(4):246–250. 28994445

[13] Fontana RJ, Engle RE, Scaglione S, et al. The role of hepatitis E virus infection in adult Americans with acute liver failure. Hepatology. 2016 Dec;64(6):1870–1880. doi:10.1002/hep.28649 27215797 PMC5115940

[14] Romano L, Paladini S, Tagliacarne C, et al. Hepatitis E in Italy: a long-term prospective study. J Hepatol. 2011 Jan;54(1):34–40. doi:10.1016/j.jhep.2010.06.017 20888660

[15] Yin X, Ying D, Lhomme S, et al. Origin, antigenicity, and function of a secreted form of ORF2 in hepatitis E virus infection. Proc Natl Acad Sci USA. 2018;115(18):4773. doi:10.1073/pnas.1721345115 29669922 PMC5939091

[16] Rydell GE, Prakash K, Norder H, et al. Hepatitis B surface antigen on subviral particles reduces the neutralizing effect of anti-HBs antibodies on hepatitis B viral particles in vitro. Virology. 2017 Sep;509:67–70. doi:10.1016/j.virol.2017.05.017 28605637

[17] Cornberg M, Wong VW, Locarnini S, et al. The role of quantitative hepatitis B surface antigen revisited. J Hepatol. 2017;66(2):398–411. doi:10.1016/j.jhep.2016.08.009 27575311

[18] Aggarwal R, Kini D, Sofat S, et al. Duration of viraemia and faecal viral excretion in acute hepatitis E. Lancet. 2000;356(9235):1081–1082. doi:10.1016/S0140-6736(00)02737-9 11009149

[19] Sridhar S, Lo SK, Xing F, et al. Clinical characteristics and molecular epidemiology of hepatitis E in Shenzhen, China: a shift toward foodborne transmission of hepatitis E virus infection. Emerg Microbes Infect. 2017 Dec 20;6(12):e115. 29259325 10.1038/emi.2017.107 PMC5750461

[20] Wang L, Liu L, Wei Y, et al. Clinical and virological profiling of sporadic hepatitis E virus infection in China. J Infect. 2016 Sep;73(3):271–279. doi:10.1016/j.jinf.2016.06.005 27343562

[21] El-Mokhtar MA, Karam-Allah Ramadan H, Abdel Hameed MR, et al. Evaluation of hepatitis E antigen kinetics and its diagnostic utility for prediction of the outcomes of hepatitis E virus genotype 1 infection. Virulence. 2021 Dec;12(1):1334–1344. doi:10.1080/21505594.2021.1922027 34002677 PMC8143225

[22] Khan AI, Salimuzzaman M, Islam MT, et al. Nationwide hospital-based seroprevalence of hepatitis A and hepatitis E virus in Bangladesh. Ann Glob Health. 2020 Mar 16;86(1):29. doi:10.5334/aogh.2574 32211299 PMC7082825

[23] Zhu FC, Huang SJ, Wu T, et al. Epidemiology of zoonotic hepatitis E: a community-based surveillance study in a rural population in China. PLoS One. 2014;9(1):e87154. doi:10.1371/journal.pone.0087154 24498033 PMC3909025

[24] Zhang J, Zhang XF, Huang SJ, et al. Long-term efficacy of a hepatitis E vaccine. N Engl J Med. 2015 Mar 05;372(10):914–922. doi:10.1056/NEJMoa1406011 25738667

[25] Zhu FC, Zhang J, Zhang XF, et al. Efficacy and safety of a recombinant hepatitis E vaccine in healthy adults: a large-scale, randomised, double-blind placebo-controlled, phase 3 trial. Lancet. 2010 Sep 11;376(9744):895–902. doi:10.1016/S0140-6736(10)61030-6 20728932

[26] Zhang J, Zhang XF, Zhou C, et al. Protection against hepatitis E virus infection by naturally acquired and vaccine-induced immunity. Clin Microbiol Infect. 2014 Jun;20(6):O397–O405. doi:10.1111/1469-0691.12419 24118636

[27] Jothikumar N, Cromeans TL, Robertson BH, et al. A broadly reactive one-step real-time RT-PCR assay for rapid and sensitive detection of hepatitis E virus. J Virol Methods. 2006 Jan;131(1):65–71. doi:10.1016/j.jviromet.2005.07.004 16125257

[28] Wen GP, Tang ZM, Wang SL, et al. Classification of human and zoonotic group hepatitis E virus (HEV) using antigen detection. Appl Microbiol Biotechnol. 2017 Oct 16;101(23-24):8585–8594. doi:10.1007/s00253-017-8526-8 29038976

[29] Myint KSA, Endy TP, Shrestha MP, et al. Hepatitis E antibody kinetics in Nepalese patients. Trans R Soc Trop Med Hyg. 2006;100(10):938–941. doi:10.1016/j.trstmh.2005.12.005 16542692

[30] Clayson ET, Myint KS, Snitbhan R, et al. Viremia, fecal shedding, and IgM and IgG responses in patients with hepatitis E. J Infect Dis. 1995 Oct;172(4):927–933. doi:10.1093/infdis/172.4.927 7561211

[31] Chandra NS, Sharma A, Malhotra B, et al. Dynamics of HEV viremia, fecal shedding and its relationship with transaminases and antibody response in patients with sporadic acute hepatitis E. Virol J. 2010;7:213. doi:10.1186/1743-422X-7-213 20815928 PMC2940811

[32] Al-Sadeq DW, Majdalawieh AF, Mesleh AG, et al. Laboratory challenges in the diagnosis of hepatitis E virus. J Med Microbiol. 2018 Apr;67(4):466–480. doi:10.1099/jmm.0.000706 29485390

[33] http://www.dongtai.gov.cn/art/2012/2/16/art_7306_1336265.html.

[34] Sarin SK, Kumar M, Eslam M, et al. Liver diseases in the Asia-Pacific region: a lancet gastroenterology & hepatology commission. Lancet Gastroenterol Hepatol. 2020 Feb;5(2):167–228. doi:10.1016/S2468-1253(19)30342-5 31852635 PMC7164809

[35] Teixeira J, Mesquita JR, Pereira SS, et al. Prevalence of hepatitis E virus antibodies in workers occupationally exposed to swine in Portugal. Med Microbiol Immunol. 2017 Feb;206(1):77–81. doi:10.1007/s00430-016-0484-8 27770276

[36] Capai L, Masse S, Gallian P, et al. Seroprevalence study of anti-HEV IgG among different adult populations in Corsica, France, 2019. Microorganisms. 2019 Oct;7(10). doi:10.3390/microorganisms7100460 PMC6843757 31623185

