# Supplementary material for: Profile of clinical characteristics and serologic markers of sporadic hepatitis E in a community cohort study
Source: Emerg Microbes Infect. 2022 Dec 18;12(1):2140613. doi: 10.1080/22221751.2022.2140613 (PMC9769141; doi:10.1080/22221751.2022.2140613)
Supplement: Supplemental Material [file TEMI_A_2140613_SM5540.pdf]

Table S1 Clinical and laboratorial profiles of 5 patients presenting only anti-HEV antibody resopnse

ID	Sample order	Sex	Age (years)	Days after onset	ALT (ULN)	HEV RNA (copies/mL)	HEV Ag (S/CO)	Anti-HEV IgM (S/CO)	Anti-HEV IgG (WU/mL)	Anti-HAV IgM (S/CO)	HBsAg (S/CO)	Anti-HBc IgM (S/CO)	Anti-HCV antibodies (S/CO)
CSY00070	1	M	71	12	87.35	neg	0.05	12.04	133.90	0.07	3.10	0.54	0.00
	2			28	2.43	neg	0.05	13.31	543.75	ND	ND	ND	ND
	3			45	1.10	neg	0.08	11.38	371.40	ND	ND	ND	ND
GAF10640	1	F	66	3	2.69	neg	0.04	2.48	0.99	0.18	0.30	0.11	0.05
	2			22	0.80	neg	0.05	0.03	6.32	ND	ND	ND	ND
GAF19088	1	F	34	6	28.74	neg	0.01	13.35	9.99	0.01	0.01	0.00	0.01
	2			24	1.29	neg	0.02	13.84	45.37	ND	ND	ND	ND
	3			39	0.51	neg	0.03	13.44	23.99	ND	ND	ND	ND
	4			136	0.29	neg	0.01	0.46	13.78	ND	ND	ND	ND
GFA07693	1	M	54	4	3.45	neg	0.07	2.59	0.04	0.33	0.11	0.00	0.01
	2			73	4.95	neg	0.12	6.38	0.17	ND	ND	ND	ND
GTY22559	1	F	59	4	2.63	neg	0.01	1.65	0.20	0.04	0.02	0.01	0.01
	2			21	2.51	neg	0.02	1.25	1.16	ND	ND	ND	ND

ND: not done; neg: negative for HEV RNA detection. ULN: the upper limit of normal. WU/mL: WHO units per milliliter. S/CO: signal to cutoff.

Table S2 Characteristics of male and female hepatitis E patients

Characteristic	Male (n=69)	Female (n=29)	P value
Age (years), mean±SD	57.38±14.17	59.97±12.42	0.266
Days after onset when presentation, mean±SD	6.10±3.42	4.83±2.58	
Length of symptomatic course (days), mean±SD	65.13±43.90	43.00±25.80	0.032
ALT (ULN), mean±SD	43.16±34.45	35.29±31.06	0.190
Positive for HEV antigen, n (%)	64 (92.75)	24 (82.76)	0.136
HEV RNA (log10, copies/mL), mean±SD	5.25±2.00	4.25±2.30	0.047
Positive for HEV RNA, n (%)	60 (86.96)	21 (72.41)	0.083
Positive for anti-HEV IgM, n (%)	68 (98.55)	29 (100.00)	0.515
Positive for anti-HEV IgG (WU/mL) when presentation, mean±SD	79.79±184.01	65.61±145.98	0.870
Positive for anti-HEV IgG-R, n (%)	40 (57.97)	20 (68.97)	0.308
Positive for anti-HAV IgM, n (%)	2 (2.90)	0 (0.00)	0.354
Positive for HBsAg, n (%)	23 (33.33)	12 (41.38)	0.448
Positive for anti-HBc IgM, n (%)	4 (5.80)	3 (10.34)	0.425
Positive for anti-HCV antibodies, n (%)	0 (0.00)	0 (0.00)	

*Plus-minus values are the mean ± SD. ALT: alanine aminotransferase. ULN: the upper limit of normal. WU/mL: WHO units per milliliter. Difference between male hepatitis E patients and female hepatitis E patients was calculated with the Mann-Whitney U test.

HEV: hepatitis E virus. Anti-HEV IgG-R: a ≥4-fold rise in anti-HEV IgG level. HBsAg: the surface antigen of hepatitis B virus. Anti-HBc IgM: anti-core protein of hepatitis B virus IgM. Anti-HAV IgM: anti-hepatitis A virus IgM. Anti-HCV antibodies: anti-hepatitis C virus antibodies. S/CO: signal to cutoff.

Table S3 Distribution of ALT levels in different groups of patients

ALT levels (ULN)	All	Hepatitis E	Other viral hepatitis
2.5≤ALT<3	1193 (29.03%)	2 (2.04%)	165 (22.79%)
3≤ALT<4	1155 (28.10%)	1 (1.02%)	173 (23.90%)
4≤ALT<5	484 (11.78%)	3 (3.06%)	85 (11.74%)
5≤ALT<6	310 (7.54%)	2 (2.04%)	54 (7.46%)
6≤ALT<7	199 (4.84%)	2 (2.04%)	44 (6.08%)
7≤ALT<8	103 (2.51%)	1 (1.02%)	28 (3.87%)
8≤ALT<9	100 (2.43%)	2 (2.04%)	19 (2.62%)
9≤ALT<10	63 (1.53%)	3 (3.06%)	15 (2.07%)
10≤ALT<15	188 (4.57%)	10 (10.20%)	48 (6.63%)
ALT≥15	315 (7.66%)	72 (73.47%)	93 (12.85%)
Total	4110	98	724

Other viral hepatitis: active hepatitis that were positive for hepatitis A, hepatitis B, or hepatitis C and negative for hepatitis E; ULN: the upper limit of normal. ALT: alanine aminotransferase.

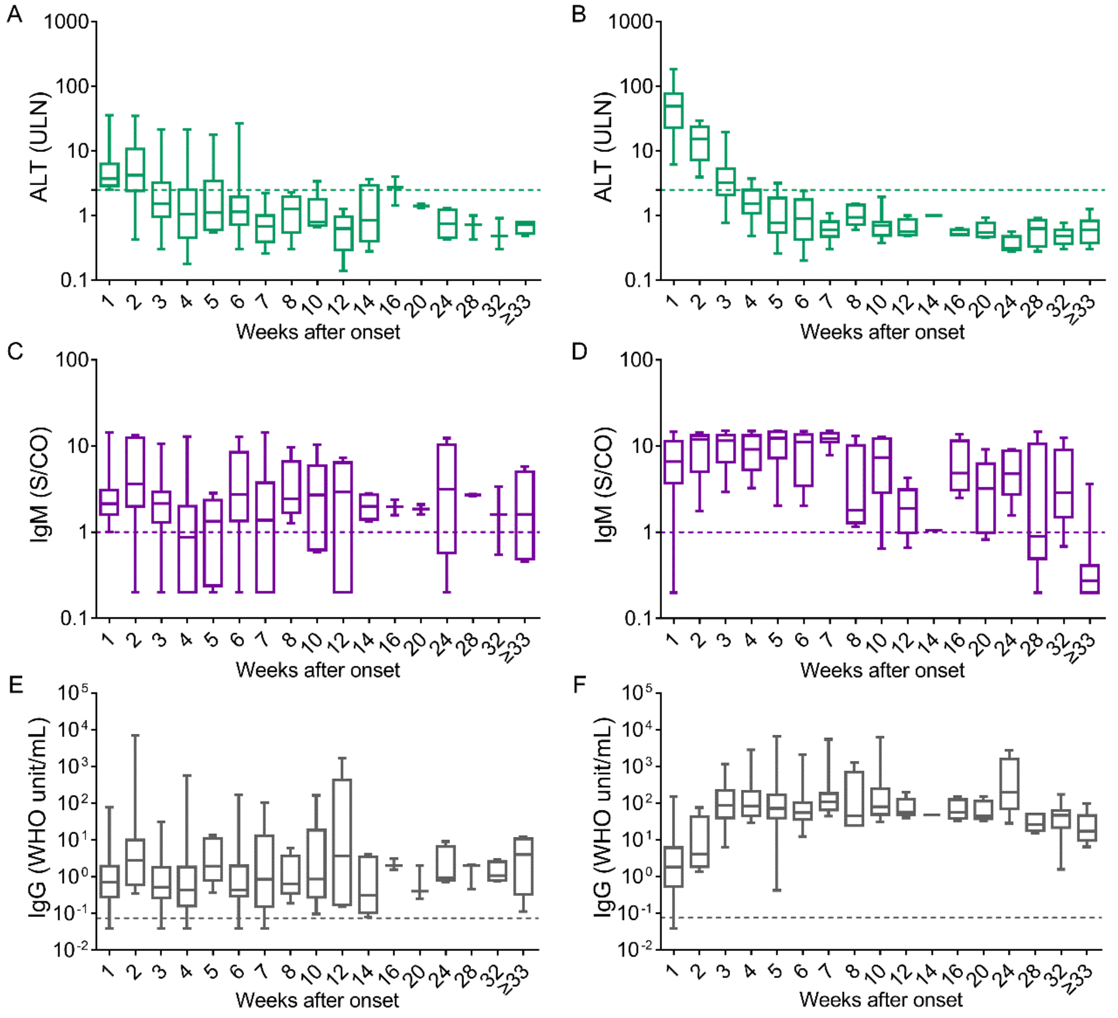

Figure S1 Dynamics of ALT and anti-HEV antibodies in 69 single-anti-HEV IgM positive active hepatitis patients (left panel, A, C, E) and 47 typical hepatitis E patients (right panel, B, D, F) with all 4 HEV-related markers during disease progression. Serum samples were collected from 69 single-anti-HEV IgM positive patients within the 1st week of symptom onset (n=59) and in the following intervals after symptom onset, the 2nd week (n=10), 3rd week (n=31), 4th week (n=31), 5th week (n=7), 6th week (n=17), 7th week (n=12), 8th week (n=6), 9 to 10 weeks (n=6), 11 to 12 weeks (n=5), 13 to 14 weeks (n=4), 15 to 16 weeks (n=2), 7 to 20 weeks (n=2), 21 to 24 weeks (n=4), 25 to 28 weeks (n=2), 29 to 32 weeks (n=3), and ≥33 weeks (n=4), respectively. Serum samples were collected from 47 typical hepatitis E patients within the 1st week of symptom onset (n=43) and in the following intervals after symptom onset, the 2nd week (n=9), 3rd week (n=27), 4th week (n=19), 5th week (n=16), 6th week (n=15), 7th week (n=9), 8th week (n=5), 9 to 10 weeks (n=7), 11 to 12 weeks (n=5), 13 to 14 weeks (n=1), 15 to 16 weeks (n=4), 17 to 20 weeks (n=5), 21 to

24 weeks (n=5), 25 to 28 weeks (n=7), 29 to 32 weeks (n=7), and ≥33 weeks (n=7). The levels of ALT (A, B), anti-HEV IgM (C, D), and anti-HEV IgG (E, F) in the different serum sample intervals are shown as range (whiskers), interquartile (boxes) and median (line within the boxes) values. The dotted lines represent the cutoff levels of corresponding markers. IgM: anti-HEV IgM; IgG: anti-HEV IgG. ALT: alanine aminotransferase. ULN: upper limit of normal. S/CO: signal to cutoff.

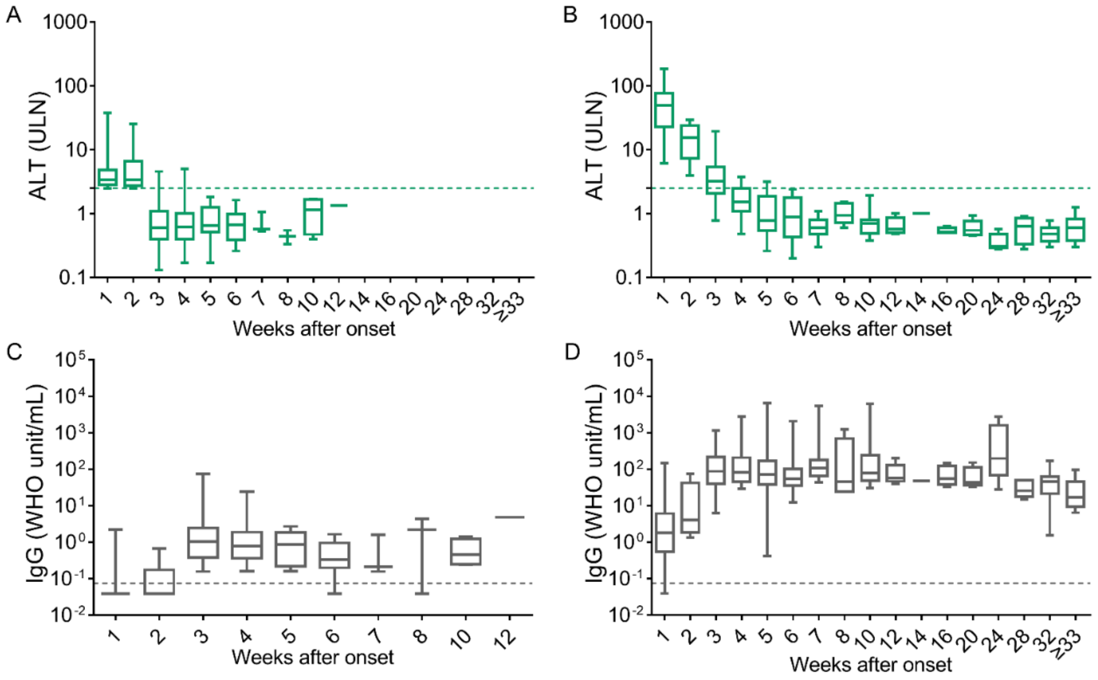

Figure S2 Dynamics of ALT and anti-HEV IgG during the illness of progression in 368 patients with single-anti-HEV IgG-R (left panel, A, C) and 47 typical hepatitis E patients (right panel, B, D). Serum samples were collected from patients with single-anti-HEV IgG-R within the 1st week of symptom onset (n=349) and in the following intervals after symptom onset, the 2nd week (n=19), 3rd week (n=136), 4th week (n=196), 5th week (n=23), 6th week (n=6), 7th week (n=3), 8th week (n=2), 9 to 10 weeks (n=4), and 11 to 12 weeks (n=1), respectively. Serum samples were collected from 47 typical hepatitis E patients within the 1st week of symptom onset (n=43) and in the following intervals after symptom onset, the 2nd week (n=9), 3rd week (n=27), 4th week (n=19), 5th week (n=16), 6th week (n=15), 7th week (n=9), 8th week (n=5), 9 to 10 weeks (n=7), 11 to 12 weeks (n=5), 13 to 14 weeks (n=1), 15 to 16 weeks (n=4), 17 to 20 weeks (n=5), 21 to 24 weeks (n=5), 25 to 28 weeks (n=7), 29 to 32 weeks (n=7), and ≥33 weeks (n=7). The levels of ALT (A, B), and anti-HEV IgG (C, D) in the different serum sample intervals are shown as range (whiskers), interquartile (boxes) and median (line within the boxes) values. The dotted lines represent the cutoff levels of corresponding markers. IgG: anti-HEV IgG. ALT: alanine aminotransferase. ULN: upper limit of normal.

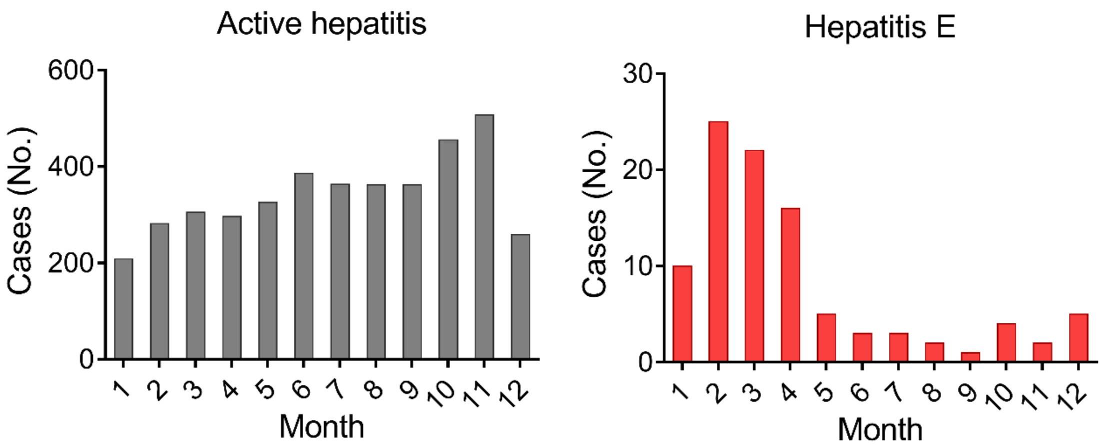

Figure S3 Seasonal distribution of active hepatitis and hepatitis E. Most hepatitis E cases occurred sporadically, with ≤3 cases detected in a village within a month and 5 cases detected in a village in March, 2013. Most (74.49%) of hepatitis E cases occurred from January to April.

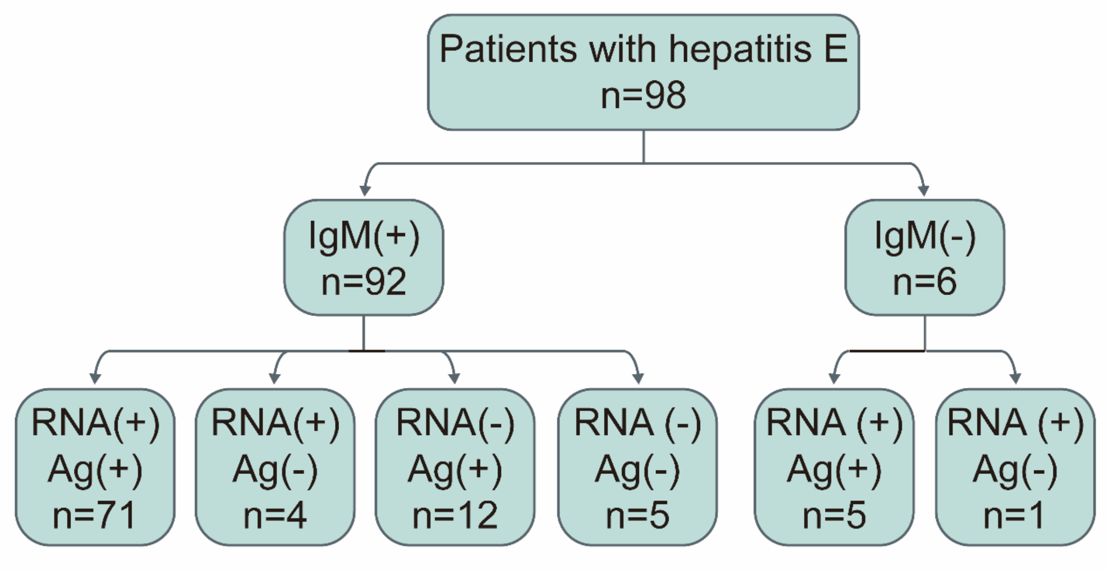

Figure S4 A schematic of anti-HEV IgM (IgM), HEV RNA (RNA), and HEV antigen (Ag) conducted on the first serum samples collected from hepatitis E patients.
